# The healing capacity and osteogenesis pattern of demineralized dentin matrix (DDM)-fibrin glue (FG) compound

**DOI:** 10.1038/s41598-023-40258-7

**Published:** 2023-08-12

**Authors:** Jibo Bao, Xunan Fu, Yirong Wu, Shengyin Yang, Xiaobin Ren, Xingchen Fang, Quan Yuan, Zhigang Xie, Dutmanee Seriwatanachai

**Affiliations:** 1 Department of Oral Biology, Faculty of Dentistry, Mahidol University, 6 Yothi Street, Ratchathewi, Bangkok, Thailand; 2 Department of Implantology, School and Hospital of Stomatology, Kunming Medical University, Hecheng International Community, Building C, No.1088 the Middle of Hai Yuan Road, Wuhua District, Kunming, Yunnan People’s Republic of China; 3 Yunnan Key Laboratory of Stomatology, Kunming, Yunnan People’s Republic of China; 4 Department of Chenggong Dental Clinic, School and Hospital of Stomatology, Chenggong New District, Kunming Medical University, University Town, Yuhua Street, Kunming, Yunnan People’s Republic of China; 5 Department of The Second Dental Clinic, School and Hospital of Stomatology, Kunming Medical University, Yuantong Street, Wuhua District, Kunming, Yunnan People’s Republic of China; 6 Department of The First Dental Clinic, School and Hospital of Stomatology, Kunming Medical University, Hongyun Street, Wuhua District, Kunming, Yunnan People’s Republic of China; 7 Department of Periodontics, School and Hospital of Stomatology, Kunming Medical University, Hecheng International Community, Building C, Wuhua District, Kunming, Yunnan People’s Republic of China; 8 School of Stomatology, Kunming Medical University, Chenggong District, Chunrong West Road, Kunming, Yunnan People’s Republic of China; 9 State Key Laboratory of Oral Diseases, National Clinical Research Center for Oral Diseases, West China Hospital of Stomatology, Sichuan University, Chengdu, People’s Republic of China

**Keywords:** Dental diseases, Preclinical research

## Abstract

Demineralized dentin matrix (DDM) is an osteoconductive and osteoinductive material that has been successfully used in sinus floor augmentation and alveolar ridge augmentation in clinical applications. It releases bone morphogenetic proteins (BMPs) and other growth factors, making DDM a suitable grafting material. However, the granular particle of DDM makes it difficult to anchor into the bone defect area. The aim of this study was to investigate the biological effects and osteoinductivity of the combination of DDM and Fibrin Glue (FG) at an optimal ratio on bone healing from a critical bone defect in an animal model. The mouse osteoblastic cell line (MC3T3-E1) was co-cultured with various ratios of DDM and FG to examine their effects on osteoblast proliferation and differentiation, as indicated by alkaline phosphatase (ALP) activity, osteocalcin (OC) production and mineralized nodules formation. The optimal ratio was then chosen for further study with a rabbit calvarial defective model, in which they were implanted with DDM or DDM-FG1 (1 g: 0.1 ml) and DDM-FG2 (1 g: 0.5 ml) compounds, or left blank for 2, 4, 8 and 12 weeks to investigate soft tissue and new bone regeneration. Micro-CT and histology analysis were used to evaluate the total grafting properties according to the different healing periods. The result from in vitro studies demonstrated that the ratio of 1:0.1 induced more ALP activity and mineralized nodules, while the ratio of 1: 0.5 (DDM-FG combined) induced more osteocalcin (OC) at specific time points. In the animal model, the 3D new bone volume in all DDM-FG treatment groups was significantly greater than that in the blank group at 2, 4, 8 and 12 weeks. Furthermore, the new bone volume was greater in DDM-FG2 when compared to the other groups during the early weeks of the healing period. In histological analysis, clusters of osteoblasts were formed adjacent to the DDM particles, and newly formed bone was observed in all groups, suggesting an osteoinductive property of DDM. Moreover, the greater new collagen synthesis observed at 4 weeks suggested that early bone healing was induced in the DDM-FG2 group. This study demonstrated that at an optimal ratio, the DDM-FG compound enhances osteogenic activities and bone regeneration.

## Introduction

In the recent bone augmentation, various types of bone graft materials have been applied. Among these materials, autologous bone demonstrated, which demonstrates conductibility, bone inductivity and osteogenesis performance, is considered the gold standard of bone transplantation^1^. However, granular bone grafts have the limitations of being easily dispersed and scattered from the implanted site. One solution is to combine them with a barrier membrane to achieve a space-maintain function. Nevertheless, alternative choices are limited in term of high cost and ethical aspects. Therefore, these drawbacks have led to developments of other bone substitutes as alternative grafting materials.

DDM is a type of dentin that has recently been used as a bone graft material. It is obtained from extracted teeth after being demineralized, and essentially represents demineralized dentin^2,3^. Although the matrix of dentin and bone have different structures, they have similar chemical compositions and may induce bone formation as consistently as bone matrix^4^. Both bone and dentin are mineralized tissues composed of approximately 18% collagen (most of them is type I collagen), 2% noncollagenous proteins (NCPs), and 70% hydroxyapatite (HA). DDM can release several growth factors, such as bone morphogenetic proteins (BMPs), vascular endothelial growth factor (VEGF), insulin‑like growth factor (IGF), transforming growth factor‑β (TGF-β), and basic fibroblast growth factor (FGF)^5,6^. BMPs are the most effective bone-inducing growth factors, belonging to the TGF-β superfamily, which are multifunctional cytokines. BMPs can significantly induce the different stages of the bone healing process such as the inflammatory phase, angiogenesis, the callus formation, and bone remodeling^7^. VEGF improves angiogenesis via induce effects in endothelial cells^8^. TGF-β1 can induce new bone formation in bone defect animal model^9^, and it cooperatively induces osteogenic differentiation with BMPs^10^. These identified growth factors play a crucial role in promoting cell migration to the bone defect region, proliferation and differentiation to form bone synthesizing osteoblasts^11^, in addition to promoting angiogenesis.

Fibrin glue (FG), also called fibrin sealant, generally consists of two main components: fibrinogen and thrombin. Fibrin is an activated form of fibrinogen that is activated by the thrombin, and the activated fibrin glue has a certain viscosity, which is conducive to blood coagulation and wound closure^12,13^. Fibrin is composed of linked fibrinogens, they play important roles in coagulation, inflammation, fibrinolysis, tissue remodeling, cellular and matrix interactions, and wound healing. In this study, we combined the DDM granular and the fibrin glue, resulting in the conversion of the granular bone grafting material into gelatinous sticky bone, which aided in the integration of the particle bone grafting material into the alveolar bone and anchored to the bone defect surface. The objective of this study was to investigate the optimal ratio of the DDM and FG in the compound, in order to determine the effect of DDM-FGs on mouse osteoblastic-cell proliferation and differentiation. Additionally, the compounds of DDM and DDM-FG were investigated in an animal model of the rabbits calvarial bone defects which can be evaluated for bone healing capacity and osteogenesis.

## Results

### The morphological property of the DDM and DDM-FG compounds.

**Figure 1:**
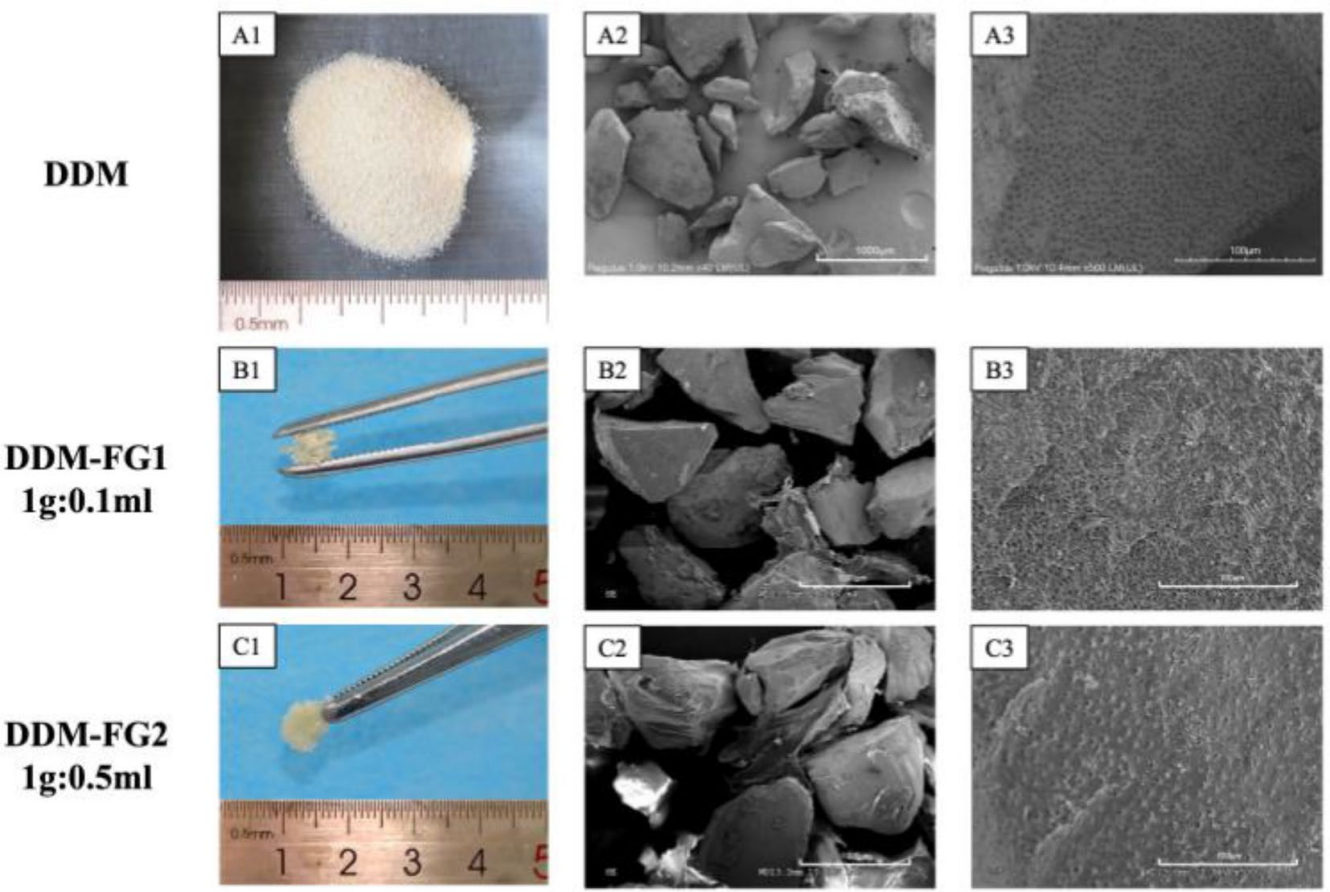
The appearance of the DDM (**A1**–**A3**), DDM-FG1 (**B1**–**B3)** and DDM-FG2 (**C1**–**C3)**. (A2, B2 and C2 with scale bar of 1000 μm; A3, B3 and C3 with scale bar of 100 μm).

The ground DDM was observed in pale yellow (Fig. 1A1). At × 40 magnification under SEM, the DDM particles appeared to be dry and loose (Fig. 1A2), meanwhile there were some layers of FG presented between and on the surface of DDM particles in DDM-FG1 and DDM-FG2 (Fig. 1B2,1C2). At × 500 magnification, most of the dentinal tubule openings of the DDM particles were typically observed and exposed (Fig. 1A3). With 0.1 ml FG, the DDM aggregate together, but the particles are loose in Fig. 1B1, at the same time, the DDM-FG2 with 0.5 ml FG, showed a sticky bone like appearance which can be held with tweezers in Fig. 1C1. DDM (Fig. 1A3), DDM-FG1 (Fig. 1B3) and DDM-FG2 (Fig. 1C3) showed obvious dentin tubular openings.

### The extent of decalcification of DDM

**Figure 2:**
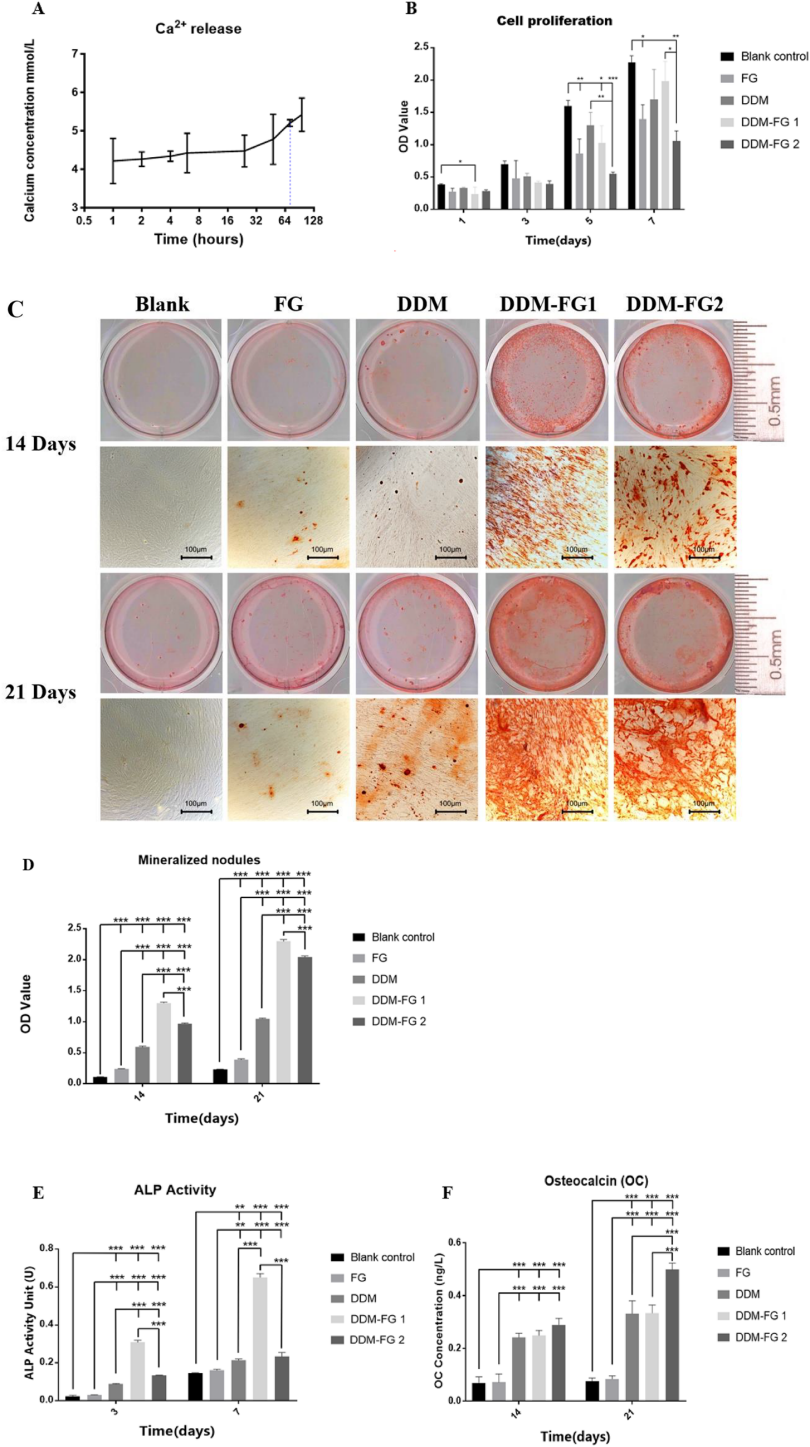
(**A**) The amount of calcium released from human dentin after decalcification. (**B**) The cell proliferation of MC3T3-E1 osteoblastic cells cocultured with different DDM-FG in transwell system. (**C**, **D**) A representation of alizarin red-staining of mineral nodules and the quantitative data of mineral nodules formed after co-cultured the MC3T3-E1 cells with different DDM-FGs for 14 and 21 days in transwell system (scale bar of 100 μm). (**E**) The ALP activity after the MC3T3-E1 cells were seeded on DDM-FGs for 3 and 7 days. (**F**) The osteocalcin secretion after the MC3T3-E1 cells were cultured with different bone graft materials for 14 and 21 days in transwell system. *Notes:* All significance was at *P* < 0.05*, *P* < 0.01** *P* < 0.001***. FG (0.1 ml), DDM-FG1 representing ratio of 1 g DDM with 0.1 ml FG and DDM-FG2 representing ratio of 1 g DDM with 0.5 ml FG.

After 72 h decalcification, calcium was found in supernatant at 5.19 mmol/L, the released calcium from DDM kept increasing after 72 h. The release of calcium from DDM confirmed that the DDM we used in this present study, was partially decalcified (Fig. 2A).

### Cell proliferation

The culture of MC3T3-E1 with different grafting materials in transwell system revealed a growth of MC3T3-E1 in all groups in time dependent manner. By day 7, DDM-FG1 significantly induced a higher amount of cell proliferation than the DDM-FG 2 and the FG alone. The blank control is slightly higher but not statistically different to the DDM-FG1 group. Nevertheless, the cell proliferations at day 7 in the group of DDM, FG, DDM-FG1 and DDM-FG2 were lower when compared to the blank control as shown in Fig. 2B.

### Calcium nodule detection

After different DDM-FG compounds were co-cultured with MC3T3-E1 cells in transwell system. The results of visible nodules and the staining intensity of each group were quantified in Fig. 2C,D. On day 14 and day 21, mineralized nodules of different sizes were visible in DDM group, DDM-FG1 group, DDM-FG2 group and FG group, presenting in red color. There were more in number and size of mineralized nodules in both DDM-FG1 and DDM-FG2 when compared to DDM group. The mineralized nodules were mostly formed in DDM-FG1 group (*P* < 0.01) which is higher than other groups. There are only few mineralized nodules in FG group that is similar to blank control group.

### The ALP activity

The ALP activity from MC3T3-E1 cultured in osteogenic medium with DDM, DDM-FG1 and DDM-FG2 were significantly higher when compared to the MC3T3-E1 alone at day 3 and day 7 as shown in Fig. 2E. The combination of DDM with FG was shown to significantly induce more ALP activity when compared to the DDM only. Moreover, the ALP activity was detected at the highest level in the DDM-FG1 group. FG did not alter the ALP activity at day 3 or day 7.

### Osteocalcin

The osteocalcin secretion detected at day 14 and 21, were significantly higher in DDM、DDM-FG1 and DDM-FG2 group than control and FG groups (Fig. 2F). The highest level of osteocalcin secretion was found in the DDM-FG2 on day 14 and significantly higher than DDM and DDM-FG1 on day 21 (*P* < 0.001).

### Morphometric and quantitative analysis from micro-CT

**Figure 3:**
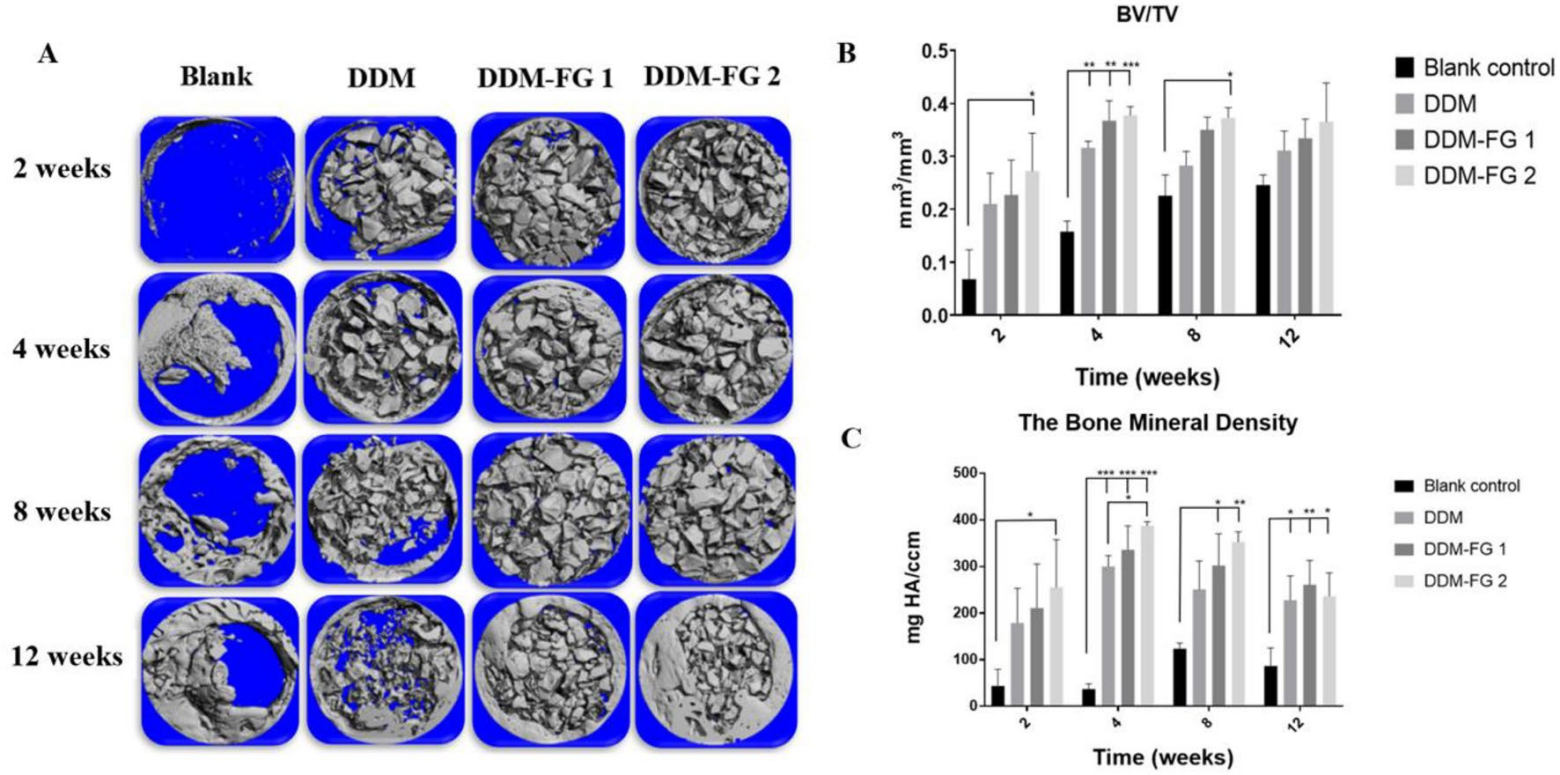
(**A**) The reconstruction 3D images of the bone defects after DDM, DDM-FG1 and DDM-FG2 were implanted for 2, 4, 8 and 12 weeks. (**B**) The BV/TV parameter and (**C**) the bone mineral density of the bone defects parameter analysis. *Notes:* All significance was at *P* < 0.05*, *P* < 0.01** *P* < 0.001***. DDM-FG1 representing ratio of 1 g DDM with 0.1 ml FG and DDM-FG2 representing ratio of 1 g DDM with 0.5 ml FG.

The Micro-CT reconstruction images (Fig. 3A) showed that there was newly bone formation from 2 to 12 weeks, however it was not fully healed the bone defective area in blank group. The newly bone was begun to form at the edge of the bone defect, which characterized as thin and irregular woven bone. For DDM group, the DDM particles seems loose and multiple small gaps were found in regenerative area especially at the early stage of the bone healing phase i.e., 2 weeks or 4 weeks (Fig. 3A). Meanwhile, the DDM particles were well attached in the DDM-FG1 and DDM-FG2 group.

The increase of BV/TV were significantly detected in DDM, DDM-FG1 and DDM-FG2 groups at 4 week compare to blank control group (*P* < 0.05) (Fig. 3B). The BV/TV showed a trend of increase as follows: blank control < DDM < DDM-FG1 < DDM-FG2 at 12 weeks. The density of the bone defects increased gradually in the early stage, while it decreased at 12 weeks (Fig. 3C). Interestingly, at the beginning stage of bone healing (2-week), the bone density in DDM- FG2 was significantly induced (*P* < 0.05). Later, the bone density of DDM, DDM-FG1 and DDM-FG2 were significantly induced (*P* < 0.001) at 4 weeks. However, the density from experimental group began to slightly decrease at 8 weeks, and the decreases in bone density from all group were more distinct at 12 weeks. Nevertheless, the bone density from all treated groups was significantly increased compared to that of the blank group at 12 weeks (*P* < 0.05). This may be the result of the DDM particles, while yielded the initial density value, while the new bone regeneration started to take over and bone remodeling occurred in later stages.

### Histological analyses

**Figure 4:**
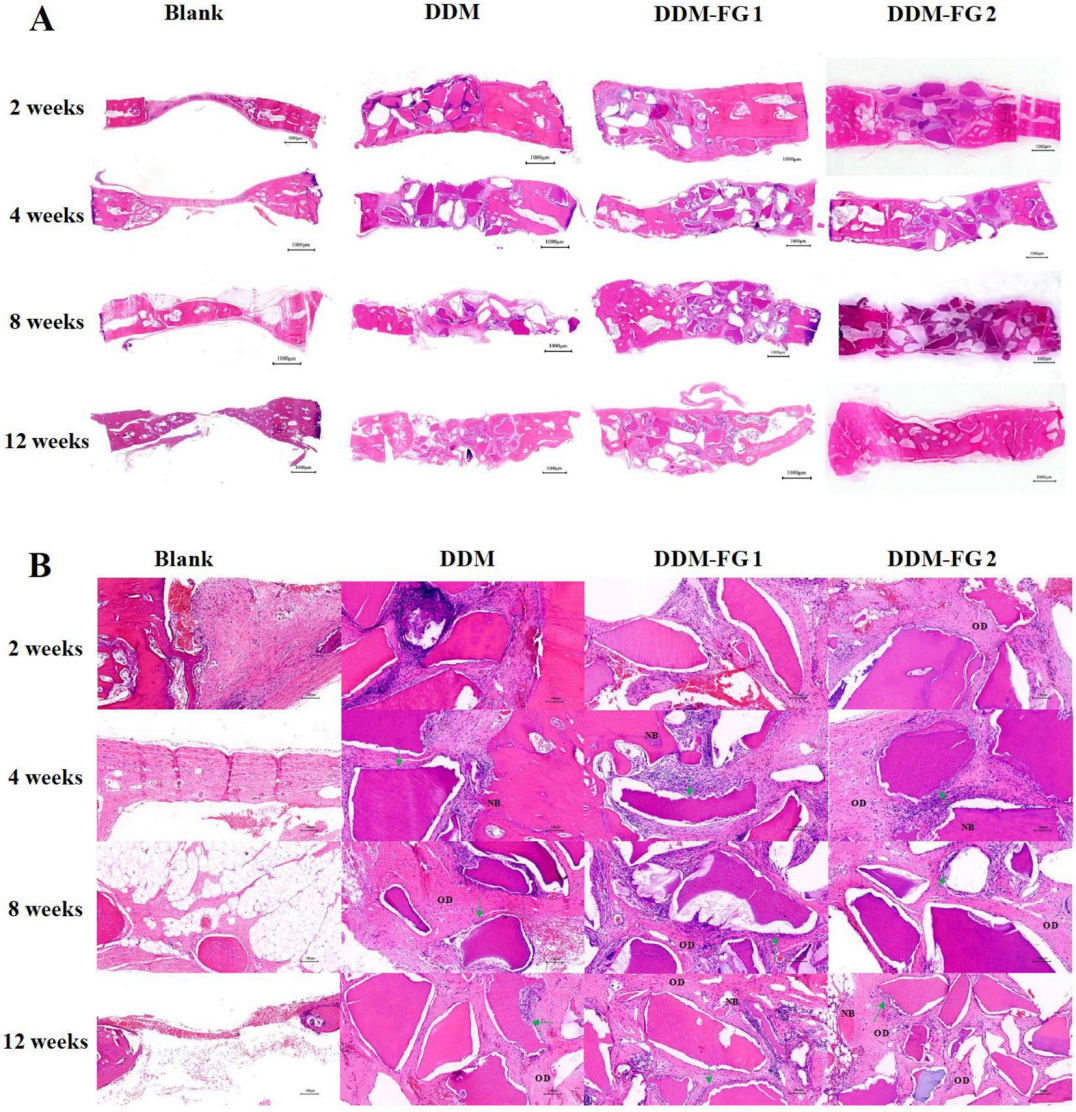
(**A**) The representative H&E staining of the bone defects which were implanted with different DDM compounds at 2, 4, 8 and 12 weeks; the scale bar = 1000 μm; (**B**) DDM particles were resorbed and surrounded by osteoid. The new bone formation was found; the scale bar = 100 μm. (NB: new bone, arrow: the adjacent osteoblasts, OD: osteoid).

In the blank group, there was no evidence of hard tissue formation in the defective area until 4 weeks, when thin mineral-stained tissue was detected at the margin of the bone defect (Fig. 4A). At 8 and 12 weeks, new woven bone visibly grew from the margin towards the center, and the thicker bone formed with the periosteum covering. However, tissue connections were not entirely filled in the blank group. The distribution of DDM particles was scattered in the DDM group, and the particles were unable to remain in the implanting position. In DDM-FG1 and DDM-FG2, DDM particles were evenly distributed in the defect with a relatively smoother surface compared to the DDM group. At higher magnification, the presence of dentin tubules was found to be gradually resorbed in all DDM groups after 4 weeks. The surrounding new bone formation was observed, with many osteocytes located in the bone lacunas (Fig. 4B). They appeared to be more newly formed bone in the DDM-FG2 group compared to the DDM and DDM-FG1 group after 12 weeks of implantation.

**Figure 5:**
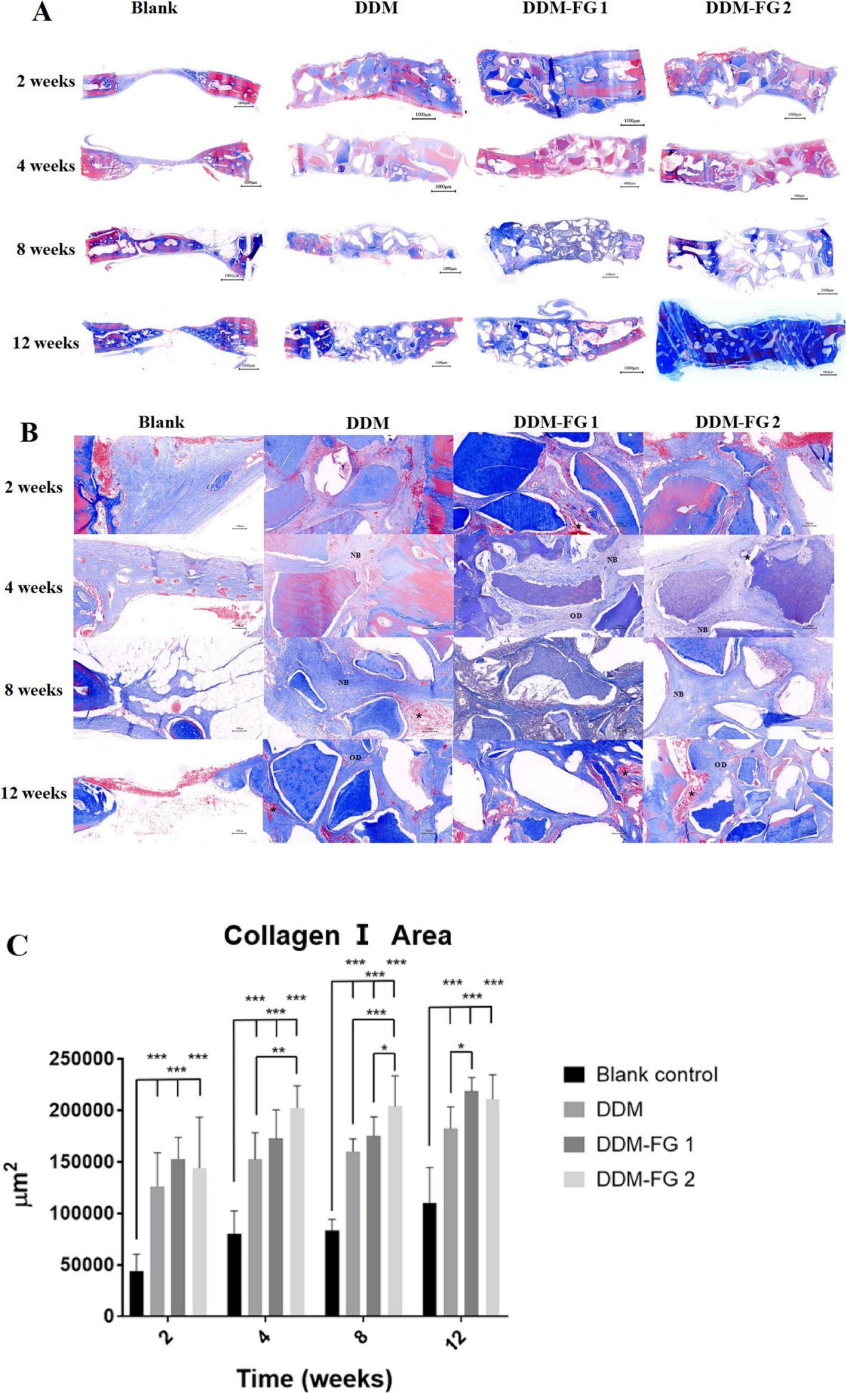
(**A**) Histological findings of Masson-stained sections (the scale bar = 1000 μm); (**B**) There were more new blood vessels and Col-I in the DDM-FG groups compare to the DDM group at 2, 4, 8 and 12 weeks, the scale bar = 100 μm**)** (NB: new bone, OD: osteoid, asterisk: blood vessels ). (**C**) The quantitative data of collagen I area of the bone defects at 2, 4, 8 and 12 weeks. *Notes:* All significance was at *P* < 0.05*, *P* < 0.01** *P* < 0.001***. DDM-FG1 representing ratio of 1 g DDM with 0.1 ml FG and DDM-FG2 representing ratio of 1 g DDM with 0.5 ml FG.

Histological findings of Masson-stained sections are similar to the HE stained sections (Fig. 5A), and there were abundant newly formed blood vessels and bone collagen I fibers in the DDM-FG1 and DDM-FG2 implantation site (Fig. 5B). The collagen I area of all groups were increased over time. It was significantly higher in the DDM, DDM-FG1 and DDM-FG2 groups than in the blank group from 2 to 8 weeks (*P* < 0.001) (Fig. 5C). The average new collagen area in DDM-FG2 group was significantly induced early at 4 and 8 weeks when compared to the DDM group (*P* < 0.01). At 8 weeks, the collagen formation in DDM-FG2 group was significantly higher than that of DDM-FG1 (*P* < 0.05).

## Discussion

The utilization of bone graft substitutes and materials in dentistry has significantly increased in recent years due to advancements in dental implant and the rising demand for the recovery of various types of bone defects. Demineralized Dentin Matrix (DDM), obtained from extracted teeth, has been used as a bone graft material in bone augmentation surgeries because of its osteoinductive and osteoconductive properties, as confirmed by several studies^14,15^. Furthermore, animal experiments have demonstrated that DDM is biocompatible and osteoinductive, comparable to decalcified bone matrix (DBM)^16^. Fibrin glue (FG) is biopolymeric material introduced as having excellent biocompatibility, controllable degrade rate, and the ability to enhance cell adhesion, improve angiogenesis. The material can be administered as a liquid to irregularly shaped sites, which adds to its surgical usefulness^17^. Given the biological characteristics of DDM particles and FG, and a need of an alternative suitable grafting material, the present study aimed to investigate the optimal condition of the combining DDM and FG for osteogenesis and bone regeneration in a rabbit calvarial defect model.

The morphological differences between the DDM, DDM-FG1, and DDM-FG2 revealed that the physical characteristics of DDM particles clearly improved with increasing FG ratio. The optimal ratio of FG maintained the naturally porous structure of DDM. In a previous study on the implantation of the DDM in a calvaria bone defect over 12 weeks, ultrastructural investigation of the interface between DDM and the surrounding new bone illustrated a network connection of the cellular processes of the osteocytes in the new bone tissue to the DDM, which extended into the dentinal tubules. This robust evidence indicated that the presence of dentine tubules provides more areas for cell attachment, facilitates substance interchange, and promotes blood vessels grown into the deep material^18^. In this study, we observed dentin tubule openings in our self-made DDM, DDM-FG1 and DDM-FG2 after effective partial demineralization. The biological effect of FG has been reported to improve osteoblast differentiation (MC3T3-E1) and cell proliferation after directly co-culture with fibrin^19^. Furthermore, in a direct seeding on osteoblasts, the concentration of thrombin dose-dependently changed the fibrin structure, providing a suitable microenvironment for osteoblast attachment and differentiation, as confirmed by level of calcium deposition, ALP activity and level of Runx2^20^. Unlike previous reports, FG alone could not effectively induce osteoblast proliferation and differentiation when compared to DDM or DDM-FGs in the present study. Meanwhile, the combination of 0.1 and 0.5 ml FG to DDM further improved cell differentiation by inducing higher ALP activity, more calcium nodules, and osteocalcin. It is possible that in our experiment’s transwell system, cells were indirectly exposed with FG and other materials, leading to a different biological response than that observed with direct cellular binding capacity.

In an animal model, the combination of calcium phosphate cement (CPC) with FG was implanted into rabbit bone defects with varied ratios. The 3D reconstruction images demonstrated that the 1:1 ratio produced the greatest bone formation^21,22^. The number of vascular sections indicated that: the 1:1 ratio was mostly effective in stimulating an angiogenesis. Thus, the combination of granular material and FG in a ratio of 1:1(g/ml) appeared to demonstrate significant bone regeneration and angiogenesis. However, in contrast to a previous animal study, our study did not find good cellular responses when using a 1:1 ratio of DDM (mg) and FG (ml) while other ratios (1:0.1 and 1:0.5) did result in good cellular responses. Moreover, the additional 0.1 and 0.5 ml of FG to DDM demonstrated supplemental effects on osteoblast differentiation, and promoted bone regeneration and angiogenesis when implanted into the rabbit calvarial bone defects. It is possible that excessive amounts of FG may not always complement the biological properties of good grafting materials, as they can completely block dentin tubules and cause the loss of the porous structure of DDM, thus interfering with cellular responses and bone regeneration.

Stable space-making and maintenance are crucial factors that could determine the success of bone regeneration^23^. The optimal particle size is essential in ensuring a proper resorption rate during bone reconstruction while maintaining defect volume for new bone ingrowth^24^. The particle size of our self-made DDM was 400–1000 μm, which provided good bone regeneration in the bone defect, similar to other previous studies^25,26^. Among other scaffold proteins, FG was proven to be a good carrier for BMP-2 release, and could prolong the BMP-2 releasing time^27,28^. Meanwhile, it is known that BMP-2 is produced and slowly secreted from DDM, with the presence of FG, it would create a suitable environment for osteogenesis and bone healing in our DDM-FGs groups than the DDM alone.

In conclusion, both DDM-FG1 and DDM-FG2 demonstrated good biocompatibility and were found to promote bone regeneration by enhancing osteogenic differentiation, improving the osteogenic microenvironment, and increasing capillarization in the bone defect area. These compounds were more practical due to their better physical properties and slower rate of degradation into the host bone. Therefore, based on the optimal ratio of the combined DDM-FGs, it enhanced osteogenic activities and bone regeneration in addition to DDM, as confirmed by the in vitro and in vivo results. This study provides new knowledge about a combination of grafting materials that can potentiate DDM as an effective biomaterial for oral surgery.

## Methods

### Preparation of DDM

In this experiment, the source of the dentin prepared for the DDM is from undecayed molar, premolar or impacted tooth from patients who came to have tooth extraction in the oral and maxillofacial surgery department, faculty of dentistry, Mahidol University. Although, the sample collection did not retain any identifying information, informed consent was obtained from all subjects and/or their legal guardian(s). All methods were carried out in accordance with relevant guidelines and regulations approved by ethic committee of the Faculty of Dentistry/ Faculty of Pharmacy, Mahidol University, Institutional Review Board (MU-DT/PY-IRB 2020/029.1007).

**Figure 6:**
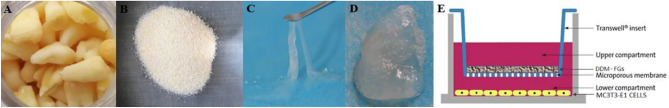
(**A**) The extracted tooth with the enamel, cementum, and soft tissues were removed; (**B**) The ground DDM particles with size of 400–1000 μm; (**C**) The freshly prepared FG and (**D**) fully gelatinized FG. (**E**) The transwell culture system used in the cell proliferation, mineral nodule and osteocalcin secretion experiments.

DDM was produced using a modified method^29^. Briefly, cold normal saline (NS) was used to wash the extracted tooth, and a dental high-speed drill was used to entirely removed the soft tissue, cementum and enamel (Fig. 6A). Then the rest dentin and pulp tissue were grinded into small particles (the particle size is 400–1000 μm) (Fig. 6B), put into ethyl alcohol/diethyl ether for 4 h at 4 °C for degreasing, then washed them twice with deionized water. In the demineralization step, 0.6 mol/L HCl was used for for 72 h at 4 °C, then repeatedly washed the particles used an ultrasonic vibrator. At last, the lyophilized DDM was packaged and sterilized by 25 kGy cobalt-60 radiation for 12 h and stored in − 20 °C.

### Fibrin glue

Porcine Fibrin Sealant Kit (Guangzhou Beixiu, China) was used in this experiment (Fig. 6C,D). The main components of FG are fibrinogen (30.0 mg/ml), and thrombin (650 IU/ml). Fibrinogen and thrombin are the main active components of the fibrin glue, which are prepared from healthy porcine plasma, which was separated, purified and virus inactivated.

### The extent of decalcification of DDM

The Ca^2+^ release was detected using a calcium detection kit (Nanjing Jiancheng Bioengineering institute) to confirm the extent of decalcification of DDM from 1 to 96 h.

### The morphological observation of the DDM and DDM-FG compounds

The DDM particles and the DDM-FG mixed compounds were observed by scanning electron microscope (SEM) (Hitachi SU8220 Japan). After freeze-drying, the specimens were fixed with 2.5% glutaraldehyde for 24 h. After washing 3 times with PBS, ethanol gradient dehydration and air drying were done. The specimens were sputter-coated with gold spraying and morphologically observed under an SEM at 12 kV.

### Co-culture of DDM-FG compounds with MC3T3-E1 cells and the osteoblast differentiation experiment

Twelve hours after seeding, MC3T3-E1 were cultured in basal medium: Dulbecco's Modified Eagle Medium (DMEM) supplemented with 10% fetal bovine serum and 1% streptomycin solution with a temperature of 37 °C, 5% CO_2_ and humidity of 70–80%. The medium was replaced every three days. The co-culture system was conducted in transwell (with pore size of 8 μm) for the cell proliferation calcium nodule and osteocalcin experiments, as shown in Fig. 6E.

### Cell proliferation

A cell count kit-8 (CCK-8, Dalian Meliun Biotechnology, China) was used to quantitatively evaluate MC3T3-E1 cell proliferation. After 500 μl cell suspension with a density of 1 × 10^4^ were grown on the transwell system () at lower chamber of 24 well plate for 4 h, the different DDM- FGs (DDM-FG1 representing ratio of 1 g DDM with 0.1 ml FG and DDM-FG2 representing ratio of 1 g DDM with 0.5 ml FG) were placed into the upper chamber. Then, after 1, 3, 5 and 7 days, the original culture medium was replaced by 500 μl DMEM with 10% FBS containing 50 μl CCK-8 reagent. After incubating at 37 °C for 4 h, 100 μl of the above solution was taken from each sample and added to a 96-well plate. Three parallel replicates were prepared. The absorbance at 450 nm was determined using a microplate reader (Muliskan, Thermo, USA). The test was independently repeated three times.

### Alkaline Phosphatase (ALP) activity

To investigate bone ALP activity, an Alkaline Phosphatase Assay Kit (Biyuntian Biotechnology, China) was used. The osteogenesis was induced by the osteogenic differentiation medium (Oricell Therapeutics, China) containing basal medium mixed with 200 μM ascorbic acid 10 mM β-glycerophosphate, 100 nM dexamethasone. Different DDM-FG compounds; DDM-FG1 representing ratio of 1 g DDM with 0.1 ml FG and DDM-FG2 representing ratio of 1 g DDM with 0.5 ml FG, were prepared and placed in 6-well plates until FG was completely gelatinized. MC3T3-E1 cells with a density of 1 × 10^4^/ml were inoculated to the surface of different DDM-FG compounds directly and cultured in 37 °C incubator. At 3 and 7 days, the cell lysis buffer was centrifuged at 1200 rpm/min at 4 °C for 25 min, and the supernatant was taken for testing. After 100 μl reaction termination solution was added to each well, the absorbance was measured at 405 nm with a microplate reader (Muliskan, Thermo, USA). The amount of ALP required for hydrolysis of para-nitrophenyl phosphate to produce 1 μmol of p-nitrophenol per minute was defined as an enzyme activity unit, alkaline phosphatase activity was calculated. The test was independently repeated three times.

### Calcium nodules measurement

2 × 10^4^/ml MC3T3-E1 cell was inoculated in transwell lower chamber in a 6-well plate, different DDM-FG compounds (DDM-FG1, 1 g DDM with 0.1 ml FG, DDM-FG2, 1 g DDM with 0.5 ml FG) were prepared in transwell upper chamber and cultured in an incubator at 37 °C (As shown in Fig. 6E). At 14 and 21 days, the different materials in the upper chamber were discarded, the cells in the lower chamber were dyed with 0.1% alizarin red for 5 min follow the Alizarin Red staining kit (Beyotime Biotechnology, China) instruction. After taking photograph of the nodules, 10% acetylpyridine was added to the 6-well plate, 500 μl/well, and incubate the solution at 37 °C for 15 min to dissolve mineralized nodules. Lastly, the incubated solutions were measured at 570 nm of absorbance with the microplate reader (Muliskan, Thermo, USA). The test was independently repeated three times.

### Osteocalcin secretion

2 × 10^4^/ml MC3T3-E1 cells were inoculated in the lower chamber of the transwell while different ratio of DDM-FG compounds was placed in the upper chamber, and cultured in the osteogenic inducing media. At 14 and 21 days, the different materials in the upper chamber were discarded, the culture medium were collected and centrifuged at 1000 rpm/min for 20 min. The supernatant was taken for osteocalcin assay using an ELISA kit (Elabscience Biotechnology, China). The intensity of the solution was immediately measured (λ = 450 nm).

### Calvaria defect surgery

The animal study protocol has been approved by the Ethical Committee of Animal Experiments, Kunming Medical University, Kunming, Yunnan Province, China (kmmu2018029). All methods were carried out in accordance with relevant guidelines and regulations, which are reported in accordance with ARRIVE guidelines. For this study, forty male adult Japanese big ear rabbit age 12 ± 3 months, weight of 3.5 ± 0.3 kg were chosen^30^. Rabbits were monitored daily throughout the study period by an accredited veterinarian. During this experiment, the rabbits were fed with food and water ad libitum. After a 4-week acclimation period, the treatment was randomized by a blinded performer who neither performed nor investigated the result, thus being “blind” to the study.

**Figure 7:**
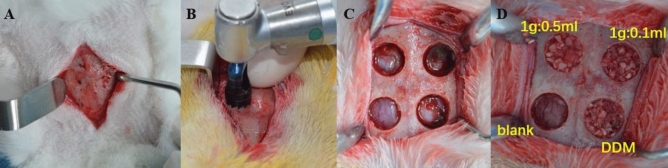
The preparation of the rabbits’ calvarial bone defects and designated grafting material replacements.

General anesthesia was induced with an intramuscular injection of 0.15 ml/kg Xylazine Hydrochloride^31^ (Jilin huamu animal health products, China). Two experienced surgeons performed all surgical procedures in 30 min, following the steps in Fig. 7. Briefly, soft tissues and calvarial periosteum were removed (Fig. 7A). A trephine bur at 6 mm diameter was used to create 4 bone defects (Fig. 7B). Blank, DDM, DDM-FG1 (1 g DDM with 0.1 ml FG) and DDM-FG2 (1 g DDM with 0.5 ml FG) were prepared (Fig. 7C), and randomly implanted into bone defect areas (Fig. 7D). An Ibuprofen suspension of 1 ml/day and amoxicillin clavulanate potassium solution 75 mg/day were mixed into the rabbits’ foods for 3 days after surgery. Each rabbit was housed in separate cages under standard laboratory conditions at an average temperature of 21 °C. All of the rabbits were survived after the surgery and were healthy until euthanasia.

Each ten rabbits were euthanized by an overdose of ketamine^32^ at 2, 4, 8 and 12 weeks. The skulls containing the surgical defect area were cut and subjected to sample preparation for µCT scan and histological analysis.

### Micro-computed tomography (micro-CT) of bone analysis

The bone samples were soaked in 4% neutral formaldehyde, and store at 20 °C. Samples were scanned using a Micro-CT scanner (SCANCO, Switzerland) with a scanning resolution of 11.4 μm, Voltage (70 kVp), Current (114 μA) at a standard rotation angle of 360° for 150 min. After scanning, data was reconstructed (μCT Ray V4.2 image processing software) and performed a quantitative analysis of all bone parameters using μCT Evaluation Program V6.6 software to examine the growth of new bone in different phases.

### Histological analysis

The 4% neutral buffered formalin solution was used to fix the samples and then stored in 70% ethanol at 4 °C before histological processing. The rabbits calvaria were soaked in Rapid Cal. Immuno™ (BBC Biochemical, USA), pH < 1, for about 6 days until they were completely decalcified. Then, the samples were rinsed for 10 min in running tap water, dehydrated with ascending graded alcohol, cleared in xylene, and embedded in paraffin. Serial sections of 5 μm thick were cut from the center of the original bone defect. Paraffin sections were stained with hematoxylin and eosin (H&E) and Masson’s-trichrome dyes and then observe under Stereo Microscope (Nikon SMZ 745 T, Japan) and Light Microscopy (AXIO Lab.A1, ZEISS, German) to investigate bone healing and collagen type I deposition, respectively. Images were then captured using NIS-Elements F 4.60.00 64-bit and ZEN 3.0 (blue edition) software. To quantify the collagen area on the Masson sections, the 3 areas of certain squares located in the middle field of each slide were calculated by Image-Pro Plus 6.0 software.

### Statistical analyses

All the data were presented as mean ± standard deviation $(\overline{x} \pm s \cdot d)$. ANOVA was used for statistical analysis, and Tukey's HSD test was used for individual comparison. The statistic software used in this experiment is Statistical Package for Social Sciences statistical software (IBM SPSS Statistics, USA). The statistical significance level was set at *p* value < 0.05.

### Ethics approval and consent to participate

Animal ethical approval for this study was obtained from the Ethical Committee of Kunming medical University (kmmu2018029). The ethic approval of the human study was granted by the Faculty of Dentistry/ Faculty of Pharmacy, Mahidol University, Institutional Review Board, Thailand (No. MU-DT/PY-IRB 2020/029.1007).

## Data Availability

The data sets used and/or analyzed during the current study are available from the corresponding author (D.S.) on a reasonable request.
